# Identification of Novel and Conserved miRNAs from Extreme Halophyte, *Oryza coarctata*, a Wild Relative of Rice

**DOI:** 10.1371/journal.pone.0140675

**Published:** 2015-10-27

**Authors:** Tapan Kumar Mondal, Showkat Ahmad Ganie, Ananda Bhusan Debnath

**Affiliations:** Division of Genomic Resources, National Bureau of Plant Genetic Resources, Pusa, IARI Campus, New Delhi-4, 110012, India; CSIR-National Botanical Research Institute, INDIA

## Abstract

*Oryza coarctata*, a halophyte and wild relative of rice, is grown normally in saline water. MicroRNAs (miRNAs) are non-coding RNAs that play pivotal roles in every domain of life including stress response. There are very few reports on the discovery of salt-responsive miRNAs from halophytes. In this study, two small RNA libraries, one each from the control and salt-treated (450 mM NaCl for 24 h) leaves of *O*. *coarctata* were sequenced, which yielded 338 known and 95 novel miRNAs. Additionally, we used publicly available transcriptomics data of *O*. *coarctata* which led to the discovery of additional 48 conserved miRNAs along with their pre-miRNA sequences through *in silico* analysis. In total, 36 known and 7 novel miRNAs were up-regulated whereas, 12 known and 7 novel miRNAs were down-regulated under salinity stress. Further, 233 and 154 target genes were predicted for 48 known and 14 novel differentially regulated miRNAs respectively. These targets with the help of gene ontology analysis were found to be involved in several important biological processes that could be involved in salinity tolerance. Relative expression trends of majority of the miRNAs as detected by real time-PCR as well as predicted by Illumina sequencing were found to be coherent. Additionally, expression of most of the target genes was negatively correlated with their corresponding miRNAs. Thus, the present study provides an account of miRNA-target networking that is involved in salinity adaption of *O*. *coarctata*.

## Introduction

*Oryza coarctata* (*Porteresia coarctata*), belonging to the family Poaceae, is an extreme halophyte and wild relative of rice which is grown in the coastal regions under high saline conditions. It is mainly found in the Eastern as well as Western coasts of India, costal region of Pakistan and Bangladesh [1]. Cytologically, it is a tetraploid (2n = 4X = 48), monotypic genus that lives throughout its life cycle in sea waters with an electrical conductivity of 30–40 dS/m [2]. It has been reported that being an extreme halophyte, it excludes the salt through unicellular trichomes called ‘salt hairs’[3]. It maintains a very low Na^+^/K^+^ ratio despite its continuous growth in artificial saline water of very high Na^+^/K^+^ ratio [3]. Genes of *O*. *coarctata*, can induce salinity tolerance in other plants including rice [4,5,6]. Thus discovery of new genes or understanding the metabolic pathways that are involved in the salinity tolerance mechanism of this halophyte will be immensely helpful in the salinity tolerance programme of rice.

Salinity is an important abiotic stress which limits the rice production world-wide and contributes 5% of the crop loss [7]. Salinity stress reduces overall plant growth by irregular cell division, defective metabolism and necrosis that ultimately results in the cell-death [8]. Out of the two types of salinity stress, while inland salinity stress is caused due to the irrigation practices with sub-standard quality of water, the coastal salinity stress occurs due to the inflow of ocean water in the cultivable land near coastal regions. Rice cultivation in India encounters both types of salinity. To overcome abiotic stresses, plants adopt several molecular mechanisms including small RNA (sRNA) mediated pathways ultimately leading to stress tolerance [9,10].

Rice, being a glycophyte, is highly susceptible to salinity stress and therefore there is an urgent need to address this problem scientifically. Salinity tolerance is a complex polygenic trait which depends on a wide range of physio-chemical processes involving various genomic resources such as QTLs, genes etc [11,12,13] including the recently discovered miRNAs [14]. MicroRNAs (miRNAs) are a group of small, non-coding, single stranded RNAs that regulate gene expression at the post-transcriptional level in response to plant abiotic stress [15,16]. Mature miRNAs are usually 21–24 nucleotides (nt) in length which are produced by RNA-induced silencing complex (RISC) to regulate the expression of genes either by inhibiting translation or by degrading specific mRNA based on the sequence complementarities between miRNA and its target mRNA [17]. Majority of the target genes of miRNAs have been found to be transcription factors or enzymes that are part of biochemical pathways involved in abiotic stress responses [18] including salinity stress response [14]. However, most of the works on the discovery of salt responsive miRNAs have been conducted on model plants such as, rice and Arabidopsis which are glycophytic in nature and hence do not possess the capability to tolerate high salinity. Only a few studies have been conducted with halophytes. For an example, novel miRNAs from halophytes such as, *Thellungiella salsuginea*, *Halostachys caspica* and *Salicornia brachiata* have been discovered recently but all these plants are dicotyledonous in nature [19,20,21,22]. Therefore, being a close wild relative of rice and monocot in nature, *O*. *coarctata* may prove to be an important reservoir of salinity tolerant genes which will be immensely helpful for improving salinity tolerance in rice plants through genetic engineering approaches.

*In silico* analysis, microarray-based approaches and high-throughput sequencing are some of the techniques that are widely used for discovery of miRNAs, yet due to technological advancements, high-throughput sequencing is the most effective method to discover the miRNAs [23,24,25,26,27]. Therefore, next generation sequencing has revolutionized the discovery of sRNA from various plant species. The availability of next generation sequencing technology provides a cost-effective, high-throughput, faster means to sequence and characterize the sRNAs of non-model and wild species as well [28,29]. Therefore, in this study, we profiled the sRNAs of salinity stressed *O*. *coarctata* by using high-throughput sequencing technology. We further analysed the interactions between miRNAs and their targets of *O*. *Coarctata* and hence made an attempt to elucidate the potential miRNA-regulated mechanisms of *O*. *coarctata* in response to salinity stress.

## Materials and Methods

### Plant materials and salinity stress

The well-rooted young saplings of *O*. *coarctata* (Roxb) Tateoka were collected from Sundarban, coastal region of Bay of Bengal, India for which no specific permission were required as they were grown under natural vegetation near the seashore. After establishing the growth in the green-house, the saplings were given salinity stress with 450 mM NaCl for 24 h. Three replicates of control and stressed samples (leaves) were harvested and kept at -80°C for future use. For control, only distilled water without any NaCl was used for 24 h treatment.

### Extraction and sequencing of small RNAs

**Fig 1:**
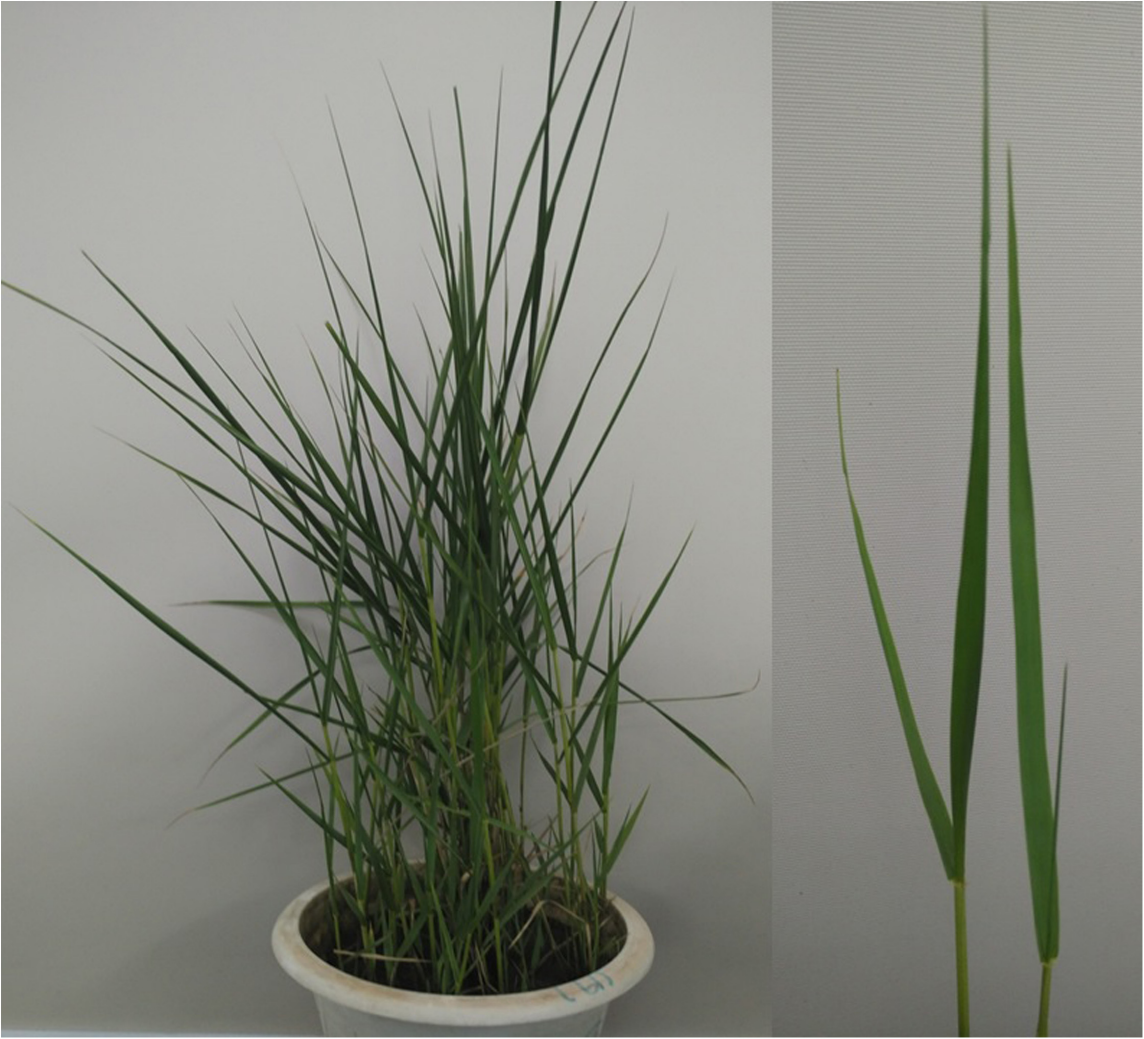
Adult saplings of *Oryza coarcta* growing under green house (left), leaf sample from two-month old sapling taken for preparation of small RNA seq library (right).

The entire work-plan is depicted in S1 Fig. Initially, total RNA was extracted from the physiologically mature young leaves of both the control and salt-treated 2 month-old saplings (Fig 1) with Trizol reagent (Invitrogen, USA) according to the manufacturer’s instructions. Both quantity and quality of total RNA were analyzed using Agilent 2100.Then 3 replicated RNA samples in equal amounts from each treatment were pooled together for sequencing. Finally, two sRNA libraries were made, one from control and another from salt-treated leaves. Small RNA libraries were prepared according to the protocol of Hafner et al [30]. Briefly, around 200ng of total RNA enriched with sRNAs was used as the starting material. Small RNA libraries for sequencing were constructed according to the Illumina protocol, outlined in “TruSeq Small RNA Sample Preparation Guide”. Small RNAs of 16–36 nt in length were purified from a 15% denaturing poly-acrylamide gel and then, 5’ and 3’ adaptors were ligated sequentially to the small RNAs. Ligated products were reverse transcribed by superscript III Reverse transcriptase (Invitrogen, USA) and resultant cDNA was enriched by PCR (15 cycles) and cleaned by using polyacrylamide gel. The library was size selected in the range of 145–160 bp in length followed by overnight gel elution and salt precipitation with glycogen, 3M sodium acetate and absolute ethanol. The prepared library was quantified by using Qubit Fluorometer, and validated for quality by running an aliquot on High Sensitivity Bioanalyzer Chip (Agilent). All sequence data have been submitted to NCBI (http://www.ncbi.nlm.nih.gov/) as Bioproject (PRJNA271658), Biosample (SAMN03275735), and Sequence Read Archive (SRA) data base under the submission accession number SRX831195.

### Identification of conserved miRNAs

Raw reads of 150 bp in length were generated by Illumina platform. Srna-workbench V3.0 ALPHA1 was used to trim the adapter sequences and perform length range filtering (minimum length 16 nt to maximum length 36 nt). Low quality and contaminant reads were eliminated from the raw reads and final clean reads were obtained by the following steps: (i) elimination of low quality reads; (ii) elimination of reads with 5’ primer contaminants; (iii) elimination of reads without 3’ primer; (iv) elimination of reads without the insert tag; (v) elimination of reads with poly A; (vi) elimination of reads < 16 nt and 36> nt in length; and (vi) summarization of the length distribution of the clean reads. Balance sequences from 16 nt to 36 nt long were used for further analysis. The small RNA tags were mapped to the rice genome using SOAP2 [31] program with no more than 2 mismatches which allowed to analyze their expression and distribution in the genome. Then sRNA tags were aligned to the miRNA precursors/mature miRNAs of rice in the miRBase 21.0 database [32] to identify the sequences and number of miRNA families in the samples. The precursors were then looked for mature miRNAs and miRNAs* manually.

### Identification of novel miRNAs

After identifying conserved miRNAs, remaining sRNA sequences were used as input in Rfam11.0 [33] to identify rRNA, tRNA, snRNA, scRNA and snoRNA as well as in the GenBank database of NCBI [34] to identify mRNA exon and intron regions which were also filtered out. We summarized all alignments and annotations before obtaining from the previous steps to avoid the duplicate mapping of same sRNA tags in more than one place. To make every unique sRNA sequence to only one annotation, we applied the following priority rule: rRNAs etc. (in which GenBank > Rfam) > known miRNAs> repeats> exons> introns [35]. Finally the remaining sequences were taken as input to identify the novel miRNAs with the software Mireap 0.2. (http://sourceforge.net/projects/mireap) to predict the secondary structure, the dicer cleavage site and the minimum free energy (MFE) of those unannotated small RNA tags which could be mapped to the rice genome. The parameters of Mireap 0.2 used are as follows: (i) minimum length of miRNA sequence is 18 nt; (ii) maximum length of miRNA sequence is 26 nt; (iii) minimum length of miRNA reference sequence is 20 nt; (iv) maximum length of miRNA reference sequence is 24 nt; (v) maximum copy number of miRNAs in the reference genome is 20; (vi) MFE for a miRNA precursor is -18 kcal/mol; (vii) maximum distance between miRNA and miRNA* is 300 nt; (viii) minimum length in the pairs of miRNA and miRNA* is 19 bp (ix) maximum bulge of miRNA and miRNA* is 8; (x) maximum asymmetry of miRNA/miRNA* duplex is 4 and (xi) flank sequence length of miRNA precursor is 20 nt. RNA sequences were considered miRNA candidates only if they satisfy all of the following criteria [36,37]. They are: (i) formation of appropriate stem-loop hairpin secondary structure; (ii) location of mature miRNA sequence in one arm of the hairpin structure; (iii) less than 2 nt mismatches of miRNAs with the opposite miRNA* sequence in the other arm; (iv) miRNA* sequences without any loop or break and (v) prediction of secondary structures with higher MFEIs, negative MFEs, and 30–70% A+U contents.

### *In silico* identification of *O*. *coarctata* miRNAs from public database

For *in silico* identification of *O*. *coarctata* miRNAs, we used initially all the pre-mature miRNAs sequence of *O*. *sativa* from miRbase 21 as input in SRA BLASTN using default parameters against the 5 transcription libraries of *O*. *coarctata*. The IDs of the libraries are: SRX248542 (Salt450 along with Submerged); SRX248538 (Control); SRX248541 (Submerged); SRX248540 (Salt700); SRX248539 (Salt450). The aligned sequences were then used as input in RNA fold (http://rna.tbi.univie.ac.at/) to predict the hairpin structure as per the procedure described above. If the sequence of pre-mature miRNA was not available then only the corresponding mature miRNA sequence was used as input for the BLASTN against the 5 libraries and only mature miRNA sequences were reported.

### Expression analysis of known and novel miRNAs

To detect differentially expressed miRNAs, we compared the level of expression of novel and known miRNAs between the control and salt-treated samples by using log_2_-ratios and scatter plots [38]. Initially, the miRNA expressions were normalized to expression of transcript per million (TPM) with the following normalization formula:-Normalized expression = (actual count of miRNAs/total count of clean reads). Subsequently, the fold-change and p-values were calculated from the normalized expression which were used to generate scatter plots and log_2_-ratio. We defined log_2_ Fold-Change ≥1 at *P*≤0.05 as up regulated and log_2_ Fold-Change ≤-1 at *P*≤ 0.05 as down regulated gene. The fold change was calculated as below:

$$\text{Fold-change}\;=\;{\text{log}}_{2}\;(\text{treatment}/\text{control})\;P-\text{value formula}$$

### Predication and validation of miRNA targets and their gene ontology analyses

Potential targets were predicted by the psRNATarget (http://plantgrn.noble.org/psRNATarget/) with default parameters [39]. In the absence of *O*. *coarcata* genome sequence, the rice TIGR genome cDNA OSA1 Release 7 (OSA1R7) was used as the reference genomic library to search the target genes.

For validation of target cleavage, total RNA (1 μg) from equally mixed 3 RNA samples was pooled to synthesize 5’- RACE-ready cDNAs with First Choice RLM-RACE Kit (Ambion, USA) according to the manufacturers instruction. A single PCR fragment was separated from the gel, cloned into the pGEM-T Easy Vector (Promega, USA) and sequenced to identify the 5’ end of the target gene. The nested and outer gene-specific primers are given in S1 Table.

For investigating the role of miRNAs in the biological processes, all the predicted targets were combined in two libraries. Then Gene Ontology (GO) enrichment analysis as well as Kyoto Encyclopaedia of Genes and Genomes (KEGG) pathway annotations were calculated [40]. Candidate targets as rice orthologos were used as input to BLAST search against rice genome with AgriGO [41] and Singular Enrichment Analysis (SEA) with the default parameters, and calibrated *P*-values were calculated. Biological function of the targets could be confirmed only when *P*-value was found to be ≤ 0.05.

### Expression analysis of mature miRNAs by quantitative real-time PCR (qRT-PCR)

To validate the high-throughput sequencing data and expression co-relation between miRNAs and respective targets, 15 miRNAs along with their target genes were selected for qRT-PCR analysis. Small RNAs (1μg) isolated from the leaves of control and salt-treated (450 mM NaCl for 24h) plants were poly-adenylated and reverse transcribed with Mir-X miRNA First-Strand Synthesis kit (Clontech) following manufacturer’s instructions. The reaction mixture (1X mRQ Buffer and 1 μl of mRQ enzyme mix), was terminated after 1 h at 85°C for 5 min. For detection of the predicted miRNAs, the cDNAs from the previous step were diluted to 20 times (1:20) in RNase-free water, and 2 μl of the diluted cDNA was used in a total reaction volume of 25 μl which was amplified by C1000 touch thermal cycler, Biorad PCR system using predicted mature miRNA sequence as sense (forward) primer and mRQ 3’ primer provided with Mir-X miRNA qRT-PCR SYBR kit (Clontech) as antisense primer (reverse). The PCR products were run on 4% agarose gel and desired fragments of required size were excised and eluted using Qiaquick gel elution kit (Qiagen). Finally, The DNA fragments were cloned in pGEM-T cloning vector provided with TA Cloning Kit (Promega) for sequencing with ABI Prism 3130xl DNA sequencer. We selected 15 primers of 3 categories: (i) 5 differentially regulated known miRNAs; (ii) 5 differentially regulated novel miRNAs and (iii) 5 randomly selected miRNAs from *in silico* analysis. All the 15 forward miRNA specific primers which were used to amplify corresponding miRNAsalong with the gene specific primers of their targets for qRT-PCR analysis are listed in S1 Table. The reverse universal primer was supplied along with the kit.

After confirming the sequences of the amplified PCR products, qRT-PCR analysis was performed in a 96-well plate using Roche 454 qPCR system (Roche, USA). Briefly, PCR reaction mixtures of 25 μl were prepared with 1 X SYBR Advantage Premix, 1 X ROX dye, 0.2 μM each of forward and reverse primers as indicated above and 2 μl of the first strand cDNA. The reactions were conducted in a 96-well PCR plate at 95°C for 2 min, followed by 40 cycles of 95°C for 10 s and 60°C for 20 s. Melting curves of the cycles were analyzed from 56°C to 95°C, with an increment of 0.5°C in every 10 s. To determine the relative expression levels of each sample, ‘comparative Ct method’ [42] was used. The threshold cycle (Ct) value of each individual reaction was normalized to the Ct value of U6 snRNA (U6 snRNA primer was provided with the kit) whose expression was found to be consistent across the conditions. All reactions were conducted with 2 biological replications each with 2 technical replications. Statistical analyses were conducted using the SAS software of JMP Genomics (SAS Institute, NC, USA).

## Results

### Properties of *O*. *coarctata* small RNAs

To identify miRNAs that are related to salinity tolerance of *O*. *coarctata*, sRNA libraries were constructed from both control sample as well as sample treated with 450 mM NaCl for 24 h. The sequencing led to the generation of 16093354 and 16729886 number of raw reads from the control and salt-treated libraries, respectively. After size selection, removing of 3’ adapter as well as different types of RNAs other than sRNAs, 14638300 number of clean reads (1140440 unique reads) from the control library and 15086882 number of clean reads (906198 unique reads) from the salt-treated library were generated (Table 1). The high-quality sRNA reads were then mapped to the rice genome sequence using SOAP 2 [31]. The number of total and unique reads that matched with miRbase sequences were found to be 369307/26275 and 303500/22876 in the control and salt-treated libraries, respectively (Table 1).

**Table 1:** Sequencing reads of control and salt-treated sRNAs libraries.

Control library	Total reads	Unique reads
**Raw reads**	16093354	-
**Clean Reads**	14638300	1140440
**Reads mapped to miRbase**	369307	26275
**Reads without annotation**	1136824	458973
**Known miRNAs**	283	-
**Novel miRNAs**	59	-
**Salt-treated library**		
**Raw reads**	16729886	-
**Clean reads**	15086882	906198
**Reads mapped to miRbase**	303500	22876
**Reads without annotation**	902177	235629
**Known miRNAs**	238	-
**Novel miRNAs**	58	-

Finally, the reads of tRNA, siRNA, intron and exon of mRNA, snoRNA, rRNA, and snRNA were removed from the total sRNA in both the libraries. Following that a total of 369307 and 303500 number of reads were mapped to the miRBase in control and salinity stressed libraries respectively (Table 1).The reads of different sRNAs are depicted in S2 Fig. It is noteworthy to mention that 1136824 in control library and 902177 reads in salt-treated library were remained unannotated indicating that these signatures might be unique sequences to *O*.*coarctata* genome. Similar results were also corroborated by Yang et al.[21].Maximum length distribution of clean reads were found to be 24 nt, followed by the 21 nt class (S3 Fig), an observation which is widely reported in different plant species[43,44,45,46] including wild species of rice [47]. Furthermore, the production of 24nt reads under salinity stressed conditions are also reported in some plant species [43]. Although, the reason of producing various sizes of sRNAs is not known, it could be due to the cleavage of precursor by DCL1 to 21 nt and by DCL3 to DCL4 to generate a range of 23 to 25 nt long miRNAs. Moreover, the generation of miRNAs that are 22nt in length from some precursors may be due to the cleavage of DCL2 [48]. Different length variations could make the suitability of RISC complex to regulate the expression of target genes either by inhibiting translation or by degrading the targets.

### Identification of known miRNAs in *O*. *coarctata*

**Fig 2:**
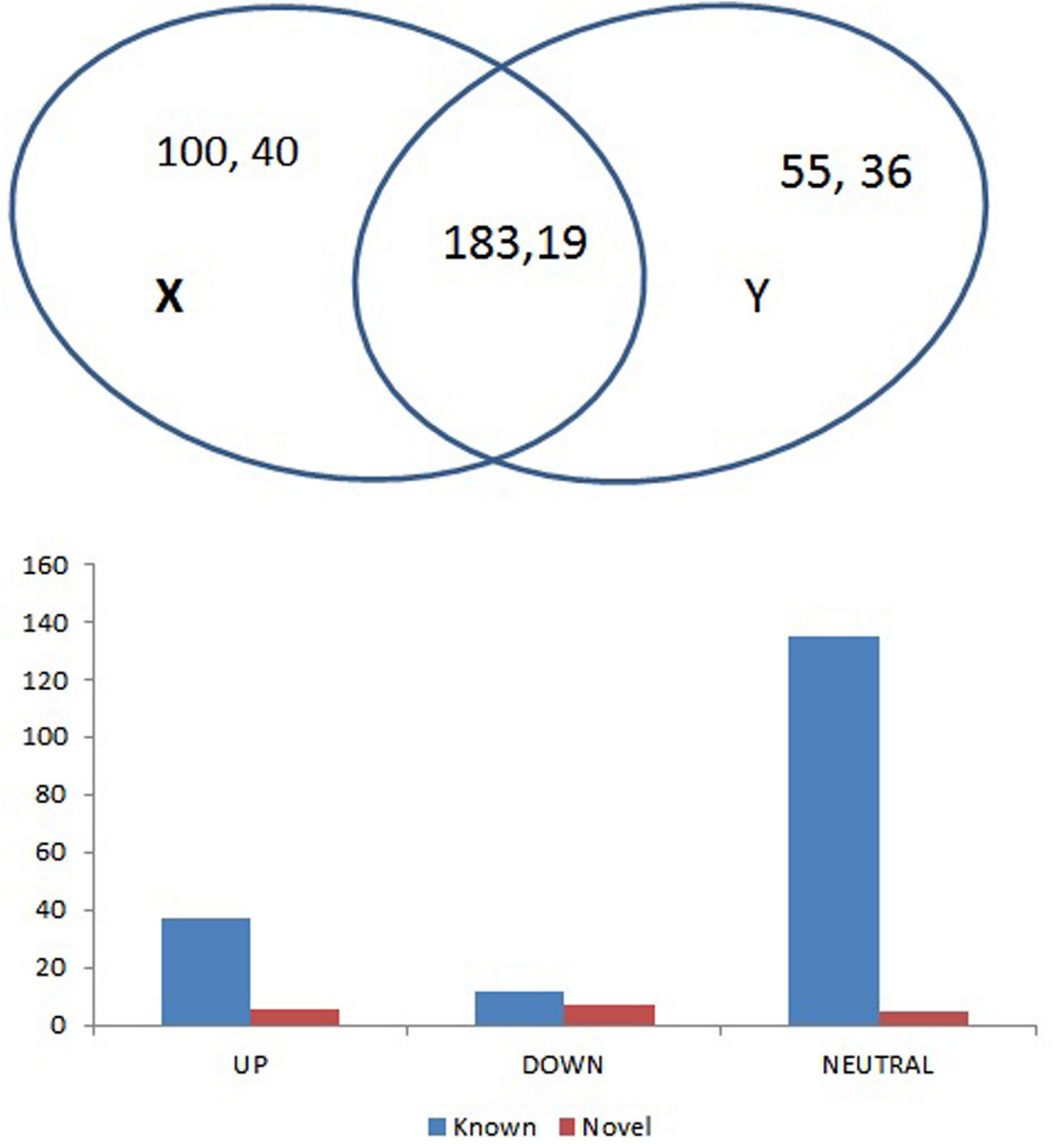
Number and expression of miRNAs of *O*. *coarctata*. A) Number of miRNAs in *O*. *coarctata*. X = Control Y = 24h salt-treated. The first numerical figure indicates known and second numerical figure indicate novel miRNAs. B) Expressions of miRNA that are found in both the libraries.

To identify the known miRNAs from sRNA sequencing, we analysed the reads of known, mature rice miRNAs from the latest version of miRBase 21.0 [32]. From the 2.04 million clean reads, a total of 338 known miRNAs were identified with a common of 183 miRNAs between the 2 libraries (Fig 2A). Based on the homology search by BLASTN, a total of 283 known miRNAs comprising 182 families were identified in the control library. Similar homology search also revealed 238 known miRNAs that comprised of 162 different miRNA families that were found in 24 h salt-treated library (Table 1). We found 7 major known miRNA families although, some miRNA families however may contain many potential members that require further validation. In the present study, the number of reads per known miRNA varied from 1 to 17092 in the control library and from 1 to 45607 in the salt-treated library. Among such miRNAs, the miR166c had the maximum reads in both the control and salt-treated libraries. Additionally, 19 miRNAs were found to have more than 100 reads and 23 miRNAs were found to have more than 10 reads in the control library. The remaining miRNAs were found to have less than 10 reads (S2 Table). Similarly, 22 miRNAs were found to have more than 100 reads and 24 miRNAs were found to have more than 10 reads in the salt-treated library. The rest of the miRNAs were infrequently sequenced (less than 10 occurrences) (S3 Table). This type of difference in expression level is widely reported in the literature [48]. While the length of known miRNAs were varied from 20 nt to 25 nt, the length of the precursor miRNAs varied from 70 nt to 101 nt.

Further, it has also been noticed that there are differences in the expression of the miRNA members. For an example, in the control library, both oco-miR2118 and oco-miR444 families had maximum of6 members each whereas, in the treated library, oco-miR166 family had maximum of 12 members followed by both oco-miR396 andoco-miR169 families, each with 8 members. Such kinds of differential expressions of the individual members within a family may be directly related to the stress response and are also reported in the literature [49]. Further, due to high sensitivity of the high-throughput sequencing chemistry, it was feasible to identify complementary miRNAs* (3p sequences) and accordingly 49 and 40 miRNAs* were detected in control and salt-treated libraries respectively. However, most miRNAs* were found to be expressed in less than 20 reads due to miRNAs* degradation during the genesis of mature miRNAs [50]. But it was seen that the expression of 3 miRNAs i.e. oco-miR166c, oco-miR 171f and oco-miR393b as well as 2 miRNAs* generated from pre-miRNAs were higher than their complementary miRNAs in control and salt-treated library respectively (S2 Table). An example was oco-miR166c in control library whose complementary oco-miR166c-3p sequence had 17092 reads, but the mature miRNA had only 2 reads. The same phenomena was also observed for some other miRNAs e.g. miR1846d, miR1853, miR1860 in *O*. *rufipogon* [47]. Thus degradation of miRNAs* is a gradual process as miRNAs* is considered to be an authentic signature for miRNA sequences because both the miRNA and miRNA* can function together to regulate gene expression.

### Identification of novel miRNAs in *O*. *coarctata*

**Fig 3:**
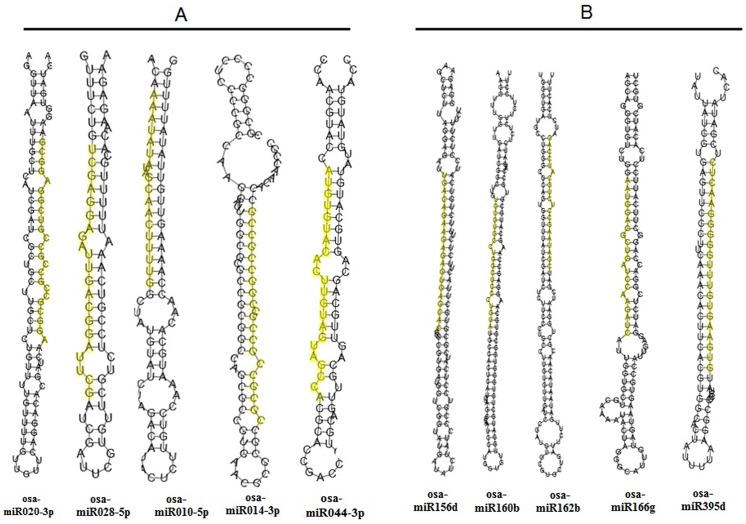
Example of predicted secondary structure of some miRNAs. A) Novel microRNAs of *O*. *coarctata*. B) Secondary structure of some miRNAs as detected by *in silico* analysis.

**Fig 4:**
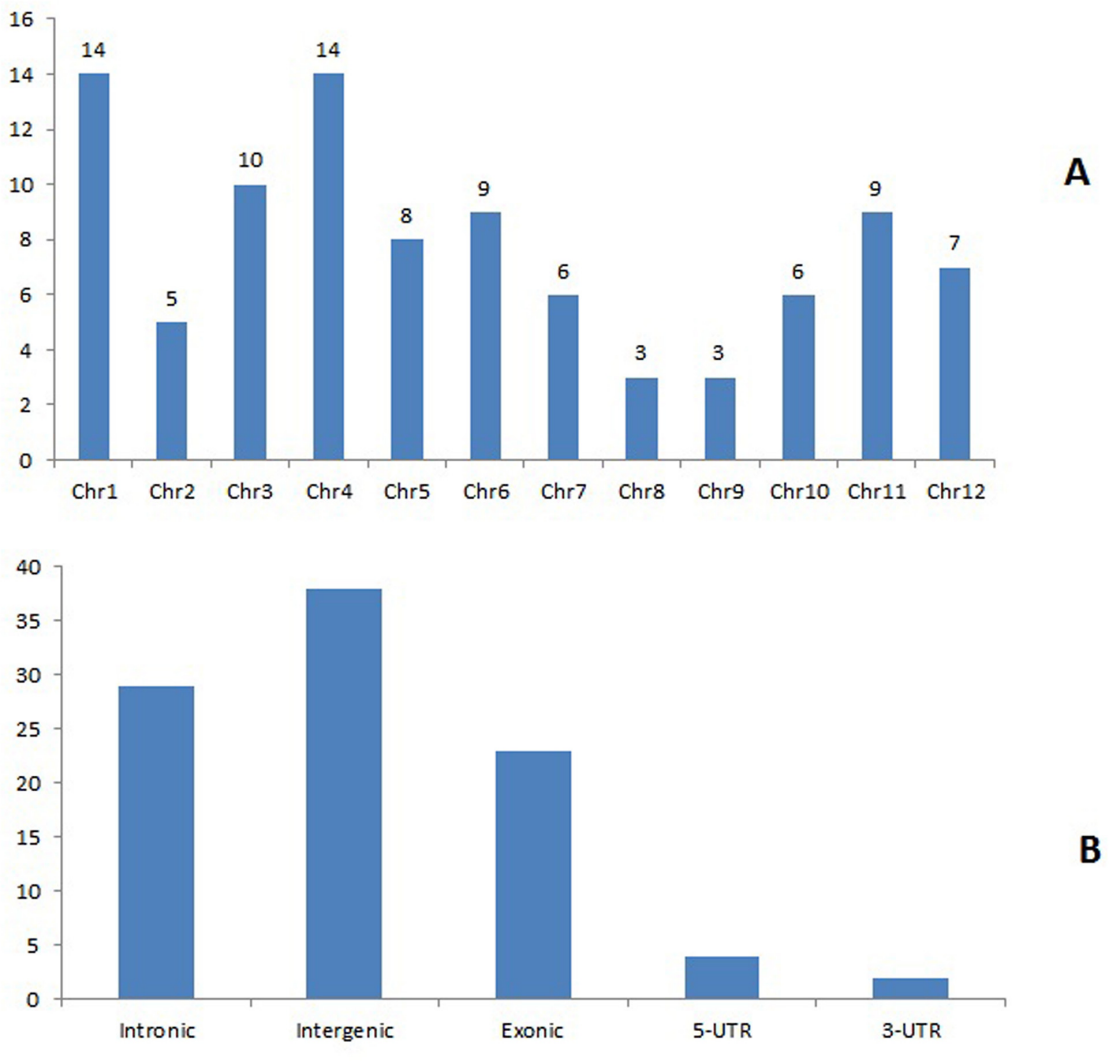
Locations and nature of novel miRNAs of *O coarctata*. A) Chromosomal distribution of miRNAs. B) Within chromosome locations of different novel miRNAs.

Stable hairpin structure is the pre-requisite for identification of novel miRNAs [35]. In the present study, to identify novel miRNAs and new members of known miRNA families, unique sRNA sequences from both the libraries were used to map on the rice genome. Secondary stem loop structures surrounding mapped sites were also predicted by special software Mireap 0.2 to identify the novel miRNAs. Additionally, binding locations of dicer enzymes and free energies were determined to evaluate these putative miRNAs. From the total 2.04 million clean reads, 95 different novel miRNAs were identified with 19 miRNAs found to be common in both the libraries. A total of 59and 58 novel miRNAs were identified in the control and salt-treated libraries, respectively (Fig 2B; Table 1; S4 and S5 Tables). It was also found that the number of novel miRNA reads showed wider variations, ranging from 1 to 22213 per miRNA in the control library and 1 to 454 per miRNA in the salt-treated library. Among them, 5 miRNAs were found to have more than 100 reads, 12 miRNAs were found to have more than 10 reads and the remaining were infrequently sequenced (less than 10 occurrences) in the control library (S4 Table). On the other hand, 4 miRNAs were found to have more than 100 reads and 15 miRNAs were found to have more than 10 reads in the salt-treated library. The remaining reads were found to occur > 10 in number (S5 Table). Stem-loop structures of some representative novel miRNAs are depicted in Fig 3. The length of the precursors varied from 61 to 101 nt with an average of 81 nt for control library (S4 Table). Similarly, length of the precursors varied from 69 to 99 nt, with an average of 81 nt for salt-treated library. The average MEF value was found to be -29.63 kcal/mol, with a range from minimum of -18.1 kcal/mol to maximum of-62 kcal/mol for control library whereas, a minimum of-18.6 kcal/mol to maximum of-47.5 kcal/mol with an average of -29.33 kcal/mol for salt-treated library (S5 Table). Locations of precursor on either strands varied between the two libraries. For an example, in the control library the number of precursors on the sense strand of the genome was found to be 39 in number,20 of them were located on the antisense strand whereas,42 and 15number of precursors were located on sense and anti-sense strand respectively in the salt-treated library. It had been seen that majority of novel miRNAs were originated from single genetic loci. However, same pre-miRNA or loci generated more than one miRNA e.g. 5 in the control and 3 in the salt-treated library. This finding is consistent with that of Chen et al. [47] who also found that one pre-miRNA sequence generated more than one miRNA in *O*. *rufipogon*, another wild rice species. Overall there were 100 and 55number of known miRNAs that were found exclusively in control and salt-treated library respectively. Similarly, there were 40 and 39number of novel miRNAs that were found exclusively in control and salt-treated library. However, 183 known and 19 number of novel miRNAs were found to be common in both the libraries (Fig 2B). We also noticed that there were differences in chromosome distribution of novel miRNAs indicating that miRNAs were originated from different positions of the genome (Fig 4A).Collectively, from both the libraries, maximum of 14 miRNAs were found to be located at 1^st^ and 4^th^chromosome each, whereas minimum of 3 miRNAs were found to be located at 8^th^ and 9^th^chromosome each. However, they differed in both the libraries. For an example, in the control library, minimum of 2 miRNAs were found on each 7^th^, 8^th^ and 12^th^ chromosome, whereas, a maximum of 10 miRNAs were located at chromosome 1. Similarly, for salt-treated library, minimum of 1 miRNA was located at chromosome 2, 8 as well as 9 while maximum 9 miRNAs were located at chromosome 1 indicating that different regulatory elements might be responsible for differential expression of miRNAs [50]. For an example, majority of them were intergenic (25 and 20miRNAsin control and salt-treated library respectively), followed by intronic miRNAs (21 and 16 miRNAs in control and salt-treated library respectively). Very few of them were found to be from untranslated regions such as from 5’-UTR (3 and 1 miRNAs in control and salt-treated library respectively) and 3’- UTR (2 miRNAs were from control library whereas none of them was found in salt-treated library). Interestingly, 8 and as high as 20number of miRNAs were found to be exonic in control as well as salt-treated library (Fig 4B; S4 and S5 Tables). Occurrence of such exonic miRNAs are also reported in rice and Arabidopsis [51]. Although exonic miRNAs are not well-studied in plants, yet Hinske et al. [52] suggested that both host genes and exonic miRNA targets may potentially be subjected to multiple layers of regulation.

### *In silico* identification of *O*. *coarctata* miRNAs from public database

Computational analysis could detect several mature miRNA sequences along with their respective pre-miRNA sequences from the 5 transcriptome libraries, available at NCBI. Among them, we could detect 124 premature miRNA sequences along with their corresponding mature miRNA sequences. While, length of the precursors varied from 46 nt to 167 nt, MFE varied from a minimum of -92.59 kcal/mol to maximum of -31.39 kcal/mol with an average of -29.30 kcal/mol. Out of the 124sequences, 56 were found in both the libraries, 14 were found in control library and 6 were found in salt-treated library. Interestingly 48 pre-miRNAs found in this analysis were not found in our deep sequencing data in either libraries (S6 Table). The stem-loop structure of few represented miRNAs are depicted in Fig 3.

Some of the known mature miRNAs are evolutionarily conserved among the various plant species. This feature has been exploited for the identification and characterization of conserved miRNAs through comparative genomics-based approaches in other plant species [53]. *O*. *coarctata* being the member of the same genus of *O*. *sativa*, we took the advantage of *in silico* analysis of some additional miRNAs particularly for this non-model plant whose genome sequence is yet to be decoded. However, the reason for not identifying the 48 miRNAs in our deep sequencing data, could be related to their low level of expression and/or spatio-temporal expression pattern, a phenomenon which has been reported by several workers [54]. Comparative genomics-based approaches through *in silico* analyses have been used to identify conserved miRNAs in several plant species [53] including rice [55].

### Differential expression analysis of known and novel miRNAs under salinity stress

**Fig 5:**
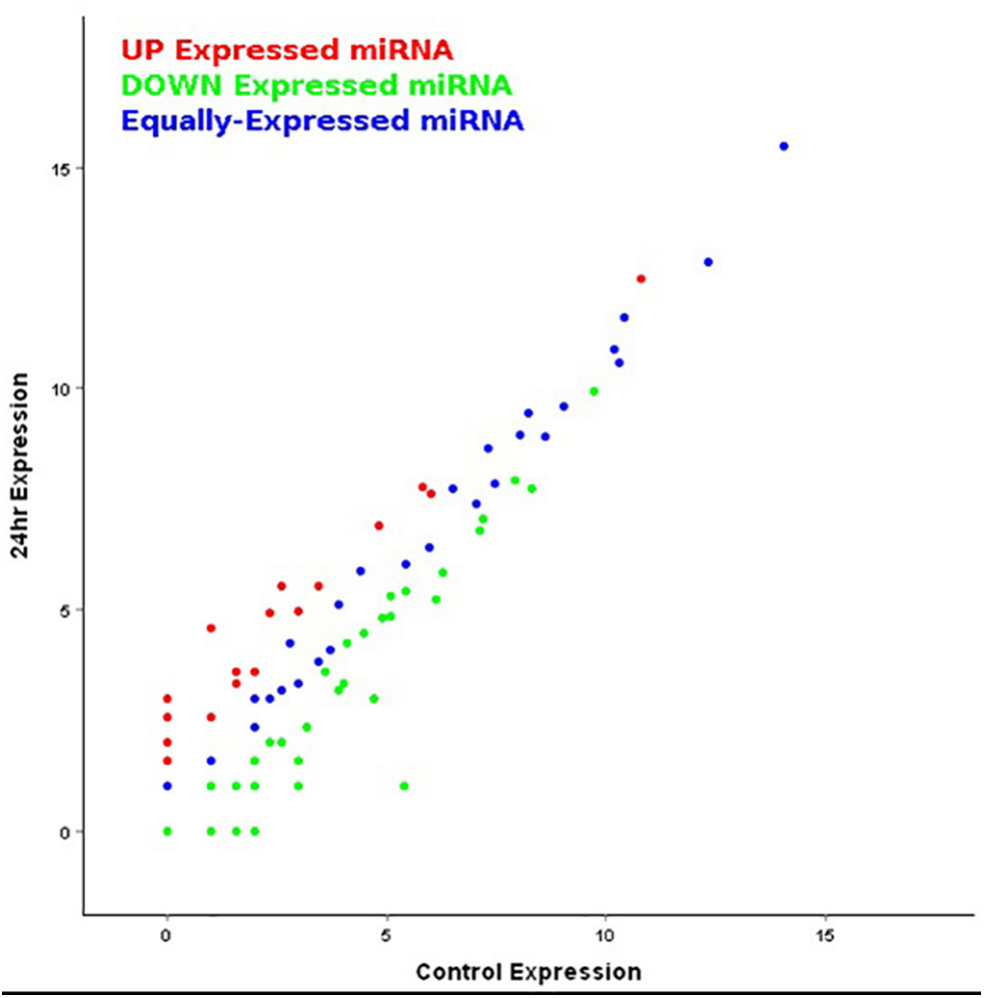
Scatter plot of novel miRNA expression level. Expression levels are normalized to TPM. Data points lower or upper the slope line represent down- or up-regulated miRNAs in panel. The changes in up- and down-regulated miRNAs are greater than 1 fold.

To identify the level of expression of known as well as novel miRNAs, we compared the expression data between the control and salt-treated libraries by using log_2_-ratios and scatter plots (Fig 5). Based on the sequencing data with log_2_-ratios value, we registered a maximum of 133as neutral in expression whereas, 37 as up-regulated (log_2_ ≥ +1, *P* ≤0.05) and 12 as down-regulated(log_2_ ≤ -1, *P ≤* 0.05) miRNA. Among the expression profiles of known miRNAs, the expression levels of oco-miR166e-3p, oco-miR169g, oco-miR528-3p, oco-miR1432-5p, oco-miR168b, oco-miR164e, oco-miR396c, oco-miR414 and oco-miR5826 had remarkable differences (S7 Table). On the other hand, among the novel miRNAs, while 5 did not change their expression, 7 each were up- and down-regulated (Fig 2B; S8 Table). The miRNAs that were expressed with greater than or less than 1.0-fold due to salt-treatment were also selected, which included 7 up-regulated and 7 down-regulated novel miRNAs. Thus depending on the expression, oco-miR028-5p, oco-miR020-3p, oco-miR044-3pandoco-miR014-3p were found to be expressed differentially (S8 Table).

### Predictions, validation and analysis of target genes for conserved and novel miRNA

Due to the perfect or near perfect complementary sites of the targets with the respective mature miRNA sequences, *in silico* prediction of targets became easy [26]. In the present study, psRNATarget (http://plantgrn.noble.org/psRNATarget/), Gene Ontology (http://www.Geneontology.org/), and the Kyoto Encyclopedia of Genes and Genomes (http://www.Genome.jp/kegg/) were used to predict and annotate various target genes of differentially expressed known and novel miRNAs. It was found that 49 known differentially expressed miRNAs of control library generated 233 target genes whereas 14 novel differentially expressed miRNAs targeted 154 genes with an average of 6.1 targets/miRNA indicating that, (i) most of the miRNAs have multiple targets, (ii) these miRNAs played multiple roles in salinity tolerance. Contrary to that, a single gene was also targeted by different miRNAs at different sites of the same cDNA and cleaved into different-sized fragments. These may be due to the fact that single targets may be involved in multiple stress response due to the cross-talk among the stresses. All the target IDs and target annotation data for the novel as well as known miRNAs are given in S9 and S10 Tables.

The number of potential targets of the known miRNAs were varied from 1 (oco-miR528-3p, oco- miR166d-5p, oco-miR5078, oco-miR529b, oco-miR5072, oco-miR162a) to 53 (oco-miR408) per miRNA. Interestingly, it was found that functions of their target genes were diverse such as those involved in growth, cell differentiation, metabolism and resistance to abiotic as well as biotic stresses. The products of target genes included transcription factors, enzymes, functional proteins and protein precursors (S9 and S10 Tables). Based on literature search [12,14,56] and functional analysis, we determined that 62 target genes were potentially related to salinity stress, hence their corresponding 22 miRNAs were therefore considered to be probable salt-related miRNAs.

**Fig 6:**
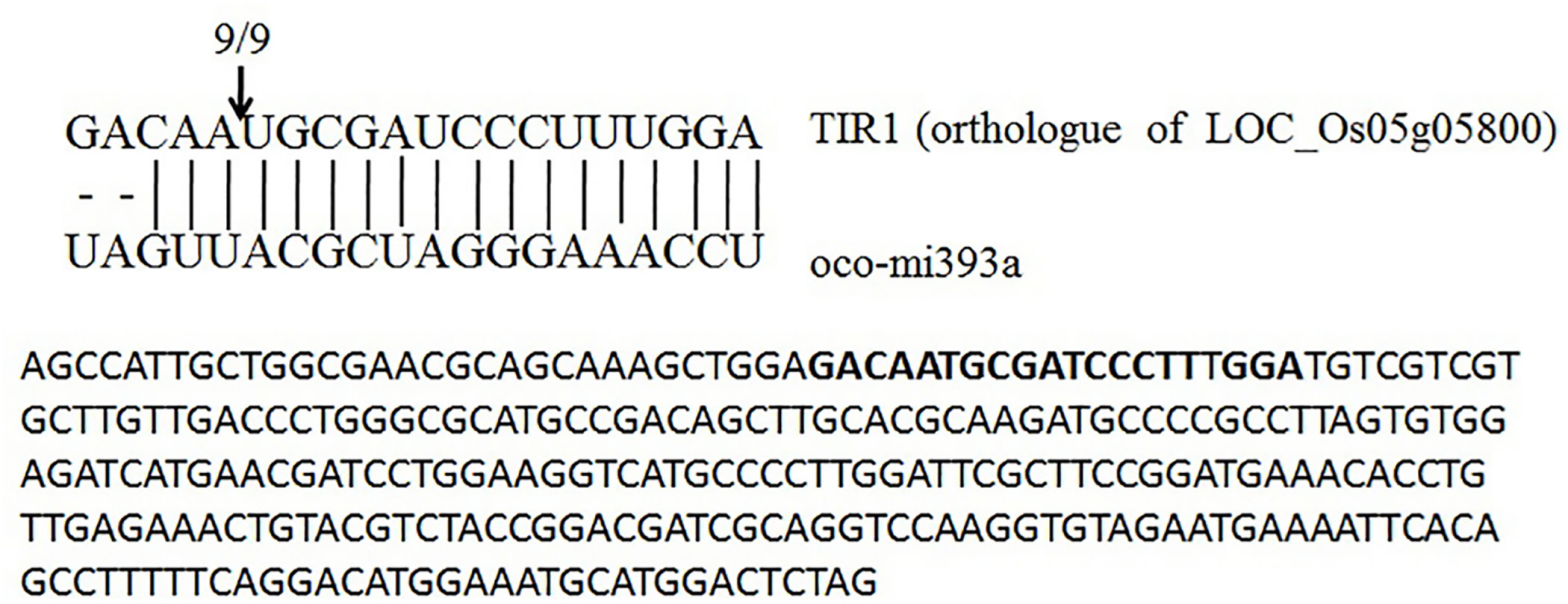
Mapping of target mRNA cleavage sites of miR393a by 5’ RACE. The target of miR393a (TIR1) encodes an auxin receptor family protein. The arrow indicates the cleavage site, and the numbers above the arrow denote the frequencies of the sequenced clones.

To validate that oca-miR393a can regulate its target mRNA expression, we amplified the predicted target gene i.e TIR1 through rapid amplification of 5′ cDNA ends (5’-RACE) from the leaf of *O*. *coarctata*. Based upon the sequence analysis, it was confirmed to be the target of osa-miR393a. Sequencing of the oco-miR393a-cleaved 5′ product of TIR1revealed a precise slice between the 10^th^ and 11^th^ nucleotide of oco-miR393a from the 5’ end (Fig 6).Using the same 5’RLM-RACE, report also indicated that miR393a cleaves its target TIR1 at complementary site (bases) in rice [57]. Therefore our results are similar to those of earlier predicted reports involving other plant systems.

**Fig 7:**
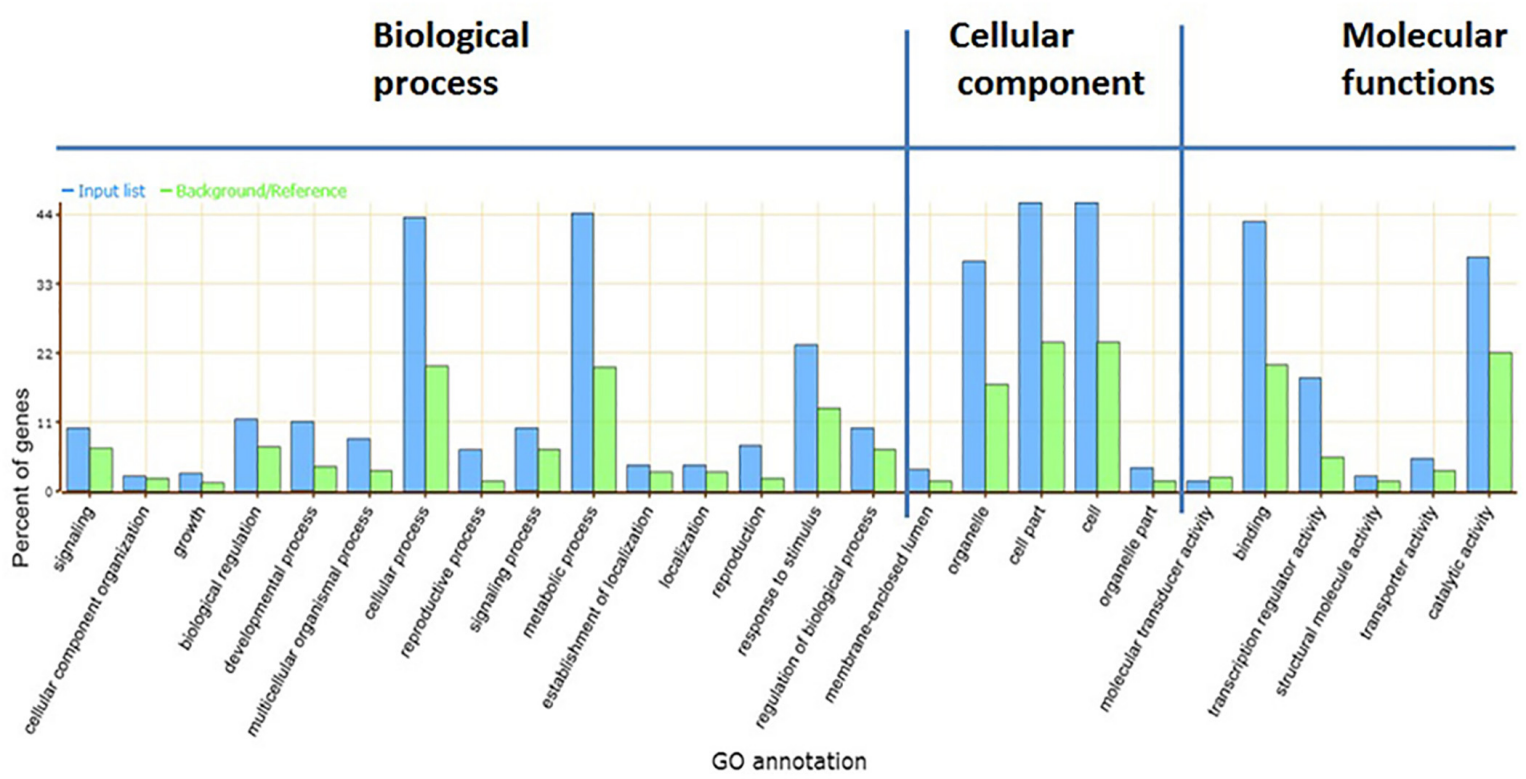
Gene ontology (GO) categories of target genes of known and new miRNA families. Categorization of miRNA target genes was performed according to the three GO domains; biological process, cellular component and molecular function.

For investigating the biological significance of the target genes regulated by salt-responsive miRNAs of *O*. *coarctata*, it was important to do GO analysis [40] i.e annotation of target gene functions, biological process as that were associated and cellular location of those gene products. In the absence of genome sequence of *O*. *coaracta*, rice orthologous genes were identified as target and used for GO analysis. Further, the targeted rice genes were subjected to SEA in AgriGO to identify enriched GOs [40]. SEA indicated enriched GO terms in a list of genes identified and analyzed by BLASTN that was searched against reference genome of *O*. *sativa* MSU 7.0 (non- TE). The number of significant GO terms was found to be 58 (Fig 7). For biological processes, these genes were classified into 15 categories and importantly 46 miRNA targets were matched with metabolic processes (GO:0008152), followed by 24 miRNA target genes that were matched with response to stimuli (GO:0050896). These results indicate that they are involved in several physiological and biochemical processes including those that are associated with salinity tolerance mechanism. Cellular Component Categories registered that the targets were located in 5 different cellular parts, of which 3 most frequent terms were found to be cell part (47), cell (46), and followed by organelle (38). On the other hand,3 over-represented GO terms out of 6 categories in Molecular Function were binding (42), catalytic activity (38) and transcription regulator activity (19) (Fig 7). In proportion with the larger number of repressed genes of *O*. *coarctata*in response to salinity stress, more enriched GO categories were identified (S4 Fig and S11 Table). In order to reduce the number of GO terms, enriched GO categories with false discovery rates (FDR) < 0.05 from AgriGO analysis were submitted to the REVIGO (REduce and Visualize GO) program [58]. Using the Uniprot database as reference with the default semantic similarity measure, it was found that biological processes associated with metabolism, cellular homeostasis, cell death, regulation of transcription and transportation were significantly over-represented among the genes of *O*. *coarctata* that were repressed by salinity stress (S5 Fig). Additionally, targets were further analysed by KEGG pathway which revealed 22 and 14 pathways in control and salt-treated libraries respectively. Important pathways that were induced in salt-treated library were phenyl propanoid biosynthesis, phenyl alanine metabolism and ubiquitine biosynthesis pathways. Some of these pathways were consistent with biological processes revealed by GO. The 5 major pathways were metabolic pathways such as starch and sucrose metabolism, phenyl propanoid metabolism, ubiquitin and other terpinoid pathways. Importantly, we found that 19 miRNA targets were involved in KEGG pathway analysis (S12 Table). As KEGG is a well-known networking database to reveal complex gene functions, the target genes were grouped as per the KEGG functional annotations to identify pathways that are regulated by miRNAs in the leaves of *O*. *coarctata*. The major enriched pathways are tabulated in S12 Table. Therefore, it appeared that the enriched miRNAs were actively involved in not only the metabolic pathway, but also in the pathways that are related to salinity tolerance.

### Expression analysis of salt-responsive miRNAs and their target genes

**Fig 8:**
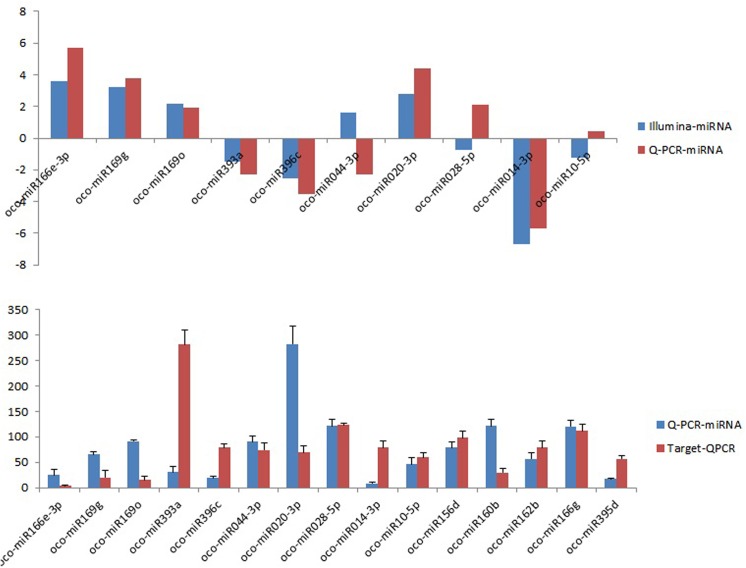
qRT-PCR analysis of the relative expression of miRNAs and targets. A) Comparison of miRNAs (fold changes) between illumina reads and qRT-PCR between control and salt-treated library. B) Relative expression of miRNAs and their targets under control and salinity stress condition. The data represents the mean values ± SD of three replicates.

To confirm and validate the data obtained from the Illumina sequencing and *in silico* analysis, qRT-PCR was done to determine the expression patterns of the 15 salt-responsive miRNAs along with the respective predicted target genes (Fig 8).It was found that the relative expression trends of 7 out of the 10 miRNAs in response to the salt treatment were the same as that predicted through both the analyses (Fig 8A). These included oco-miR166e-3p, oco-miR169g, oco-miR169o and oco-miR020-3p which showed up-regulation under salinity stress with both high-throughput sequencing as well as qRT-PCR analyses whereas, oco-miR393a, oco-miR396c and oco-miR014-3p showed down-regulation under salinity stress with both by Illumina sequencing as well as qRT-PCR analyses. On the contrary, oco-mi047-5p, oco-miR046 andoco-miR074 showed different expression patterns under salinity stress between qRT-PCR analysis and high-throughput sequencing reads. Among the target genes, oxidoreductase (LOC_Os05g41010), cullin family domain containing protein (LOC_Os05g10580) and F-box domain containing protein (LOC_Os02g49520) for oco-miR393a, oco-miR396c and oco-miR014-3p respectively were up-regulated under salt-treatment, while others such as nuclear transcription factor Y subunit (LOC_Os02g53620), histone-lysine N- methyltransferase, H3 lysine-9 specific SUVH1 (LOC_Os06g16790), auxin response factor (LOC_Os04g43910) for oco-miR169g, oco-miR020-3p, oco-miR160b respectively were down-regulated. The relative expression trends as revealed by qRT-PCR and Illumina sequencing data of 15 miRNAs and their corresponding target genes were found to be similar for 7 miRNAs and all of them had a reverse correlated expression trends with their corresponding target genes. Therefore, those 7 miRNAs i.e. oco-miR166e-3p, oco-miR169g, oco-miR169o, oco-miR393a, oco-mi396c, oco-miR020-3p and oco-miR014-3pwere considered to be potential salt-responsive miRNAs (Fig 8B). Our findings are with the concurrence of Zhao et al.[59] who also found that osa-miR169g and osa-miR169o were up-regulated under salinity stress of rice. Apart from that osa-miR396c [60], osa-miR393a [57, 61], osa-miR166 [62] are also found to be salt responsive miRNA of rice.

### Abundance of *O*. *coarctata* novel miRNAs in monocots and dicots

In the present study, we identified 95 novel miRNAs of *O*. *coarctata* and named as oco-miR01 to oco-miR93. Among these miRNAs, though we did not find any family members i.e different precursor sequences with same/similar mature miRNA sequences, yet we found 3 pairs of miRNAs(oco-miR10-3p/oco-miR10-5p, oco-miR70-3p/oco-miR70-5p andoco-miR80-3p/oco-miR80-5p) that were produced by same precursor which, we named as 5-p and 3-p based upon their occurrence in the precursor sequence. Similar observation is also reported by Feng et al. [22], who found only one family member out of 31 novel miRNAs of halophyte *Salicornia europaea*.

Further, in order to find out the abundance/presence of these novel miRNAs across the different monocot and dicot species, we selected 6 dicots and 17 monocots for BLASTN search that yielded 5 different types of miRNAs. They are: (i) miRNAs which are conserved across the all species e.g. oca-miR007-5p and oca-miR017-3p.; (ii) miRNAs that were found only in monocot i.e. monocot specific miRNAs e.g.oco-miR002-3p, oco-miR002-5p, oco-miR030-3p, oco-miR66-5p and oco-miR016-3p.; (iii) miRNAs that were found exclusively in the *Oryza* species and therefore we termed them as *Oryza* genus specific miRNAs e.g. oco-miR004-5p, oco-miR005-3p, oco-miR006-3p, oco-miR009-3p, oco-miR011-5p, oco-miR012-3p, oco-miR014-3p, oco-miR018-5p, oco-miR019-5p, oco-miR022-5p, oco-miR033-5p, oco-miR035-3p, oco-miR045-5p, oco-miR047-3p, oco-miR051-5p and oco-miR61-3p; (iv) miRNA that was found only in *O*. *coarctata*, e.g. oco-miR027-3p. and (v) finally, a large number of mixed types miRNAs that did not follow any pattern, were also found (S13 Table). To validate the above results, the precursor sequences of 10 novel miRNAs (oco-miR012-3p, oco-miR014-3p, oco-miR020-3p, oco-miR027-3p, oco-miR028-5p,oco-miR047-3p, oco-miR053-5p, oco-miR60-3p, oco-miR69-5p and oco-miR83-5p) were cloned and sequenced. While 8 of these sequences were identical to the sequences obtained from Illumina sequencing, 2(oco-miR012-3p and oco-miR047-3p) contained less than 3 mismatched nts (S13 Table). Although the reason is not known but this may happen due to the sequence assembly mistakes in Illumina sequencing. Therefore, in the present study, not only novel miRNAs of *O*. *coarctata* were discovered but also 93 miRNAs that are absent in the miRBase presently, were added to the list of rice miRNA.

## Discussion

Salinity stress is one of the important abiotic stresses that limits crop production. Plants react to salinity stress with different domains of gene regulation. Apart from various genomic resources such as transcription factors [63,64], protein coding genes [13,65] and QTLs[66], miRNAs have been found recently to be important regulator of salinity stress response[14,57].Next generation sequencing is a powerful technique to reveal the deep inside of genomic information. Recently, it has been used for identification and expression analysis of salt responsive miRNAs in several plants e.g. rice [67,68,69], broccoli [70],wheat [71], switch grass [72], sugarcane [73], Medicago [74], populus [75], barley [76] and maize [62]. However, most of them are focused on discovery of miRNAs from glycophytes or the tolerant genotypes of glycophytic plants. Recently, miRNAs have been discovered from two halophytes but both of them are dicot in nature (Table 2). Therefore, discovery of miRNAs related to salinity stress from a monocot halophyte will be extremely useful which could contribute important understanding about the molecular functions of monocot miRNAs. Therefore in the present study, we attempt to discover the miRNAs of an extreme halophyte which is also a monocot species, *O*. *coractata* by using high-throughput sequencing.

**Table 2:** miRNAs discovered by high-throughput sequencing from the different halophytes.

Plant	Nature	Conserved miRNAs	Nobel miRNAs	Reference
***Halostachys caspica***	Dicot	170	102	[21]
***Salicornia europaea***	Dicot	210	31	[22]
***O*. *coarctata***	Monocot	338	98	Present study

In the present study, leaf tissue has been taken to prepare the sRNA library. Although root is the first organ that encounters the soil related stresses, nevertheless, leaves are also an important organ where post stress effects are mainly reflected. Therefore, several workers have taken leaf as the tissue to identify salinity stress related genomic resources in rice and other plants [77,78,79,80,81]. Specific to *O*. *coarctata*, the salt glands play an important role to remove the excess salt [2]. These special unicellular structures are also found in the furrows on the adaxial surface of leaf [1,2]. Therefore, to know salt tolerant related mechanisms that are mediated by miRNAs, leaf tissue was used.

Around, 2.04 million unique reads were generated which could detect 338 known and 95 novel miRNAs from an orphan species for the first time and the number of miRNAs discovered is also higher than the other similar reports of discovery of miRNAs from halophytes (Table 2). Additionally several unannotated reads were found indicating that they are specific reads of this genome. However, the expression patterns of miRNAs varied from one to several thousand reads suggesting that there is a wide range of expression variations of miRNAs. Contradictory to the earlier reports, several salt responsive miRNAs did not show any expression here. This discrepancy suggests that these miRNAs potentially expressed in a species-specific manner under salinity stress. For example, oco-miR159b, oco-miR166c-3p, oco-miR396e-5p and oco-miR168a-5p had extraordinarily high number of reads, suggesting these miRNAs might be expressed at a higher level whereas, oco-miR161 and oco-miR393,which are well-known salt responsive miRNAs showed low abundance with less than 100 number of reads due to their lower level of expression.

Although there are few reports on *de novo* discovery of miRNAs from wild species of rice yet none of them were related to any abiotic stresses including salinity stress. For an example, Zong et al. [82] recently discovered miRNAs from various tissues of *O*. *longistaminata* by high-throughput sequencing. Similarly, earlier, Chen et al. [47] identified 512 miRNAs from vegetative as well as flowering stages of *O*. *rufipogon*. Besides, osa-mi159 and osa-mi319 were cloned from various species of *Oryza* to understand the genetic diversity among the different species of *Oryza* [83]. In this study, a number of miRNAs were identified from *O*. *coarctata*, some of which are salt-responsive and might play crucial roles in regulatory network of salinity stress response by regulating specific stress-related genes.

**Fig 9:**
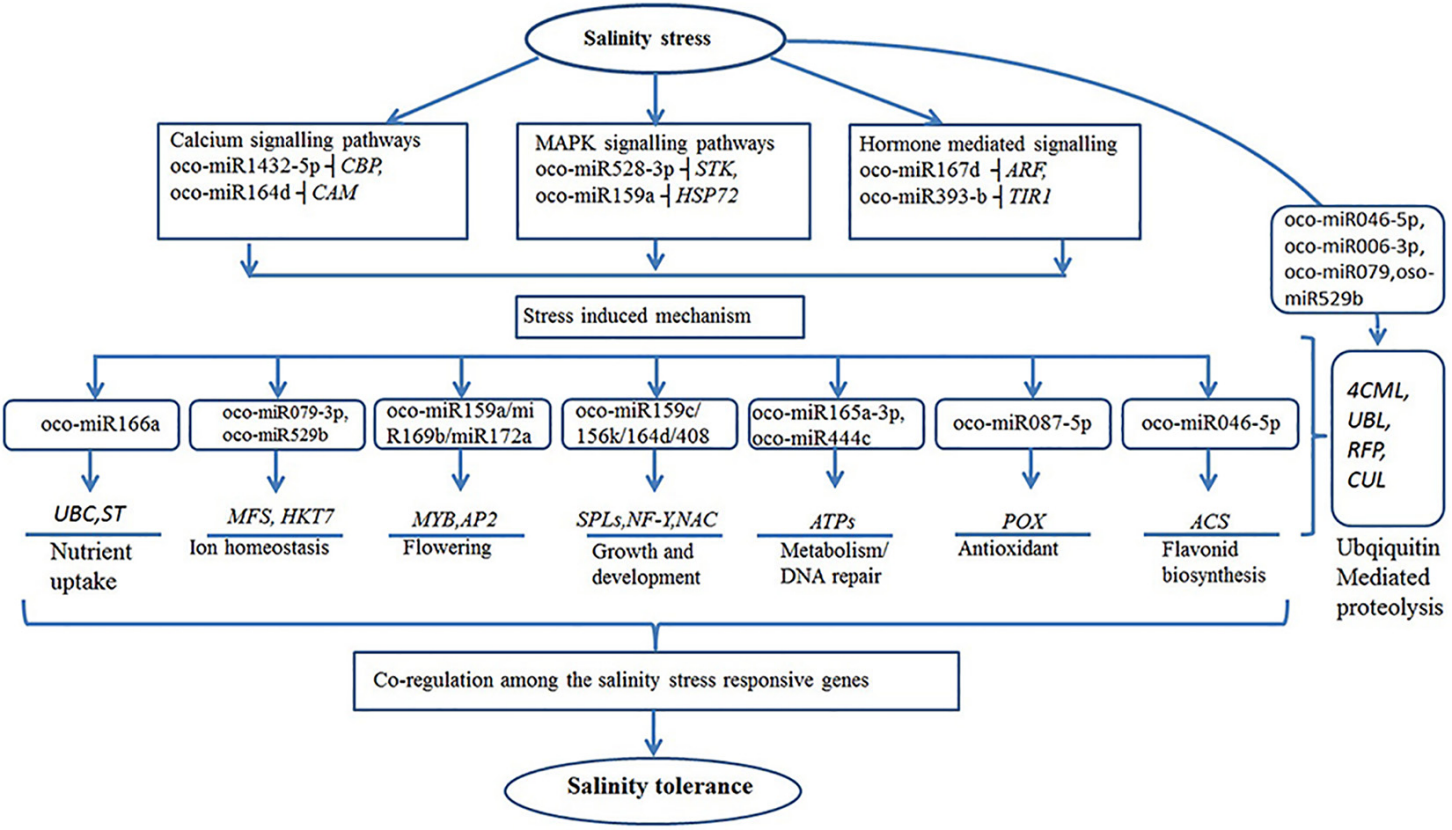
Predicted miRNA-target interaction in *O*. *coarctata*. Target genes are denoted by italics: ubiquitin-conjugating enzyme(*UBC*); Sucrose transporter (*ST*); Major facilitor super family protein (*MFS*); MYB; APETALA2 (*AP2*); SPB-like proteins(*SPL*); auxin response factor(*ARF*); nuclear transcription factor Y (*NF-Y*); NAC domain-containing proteins (*NAC*); ATP synthatase (*ATPs*); peroxidase (*POX*); Anthocyanidin synthatase (*ACS*); 4-coumarate-CoA liagase-1 (*CML*); Ubiquitin protein liagase (*UBL*); Ring Finger protein (*RFP*); Cullin-1(*CUL*); serine/threonine-protein kinase 38 (*STK*); Calcium binding protein (*CBP*); Cation transporter HKT7 (*HKT7*); Calmodulin (*CAM*).

With the present findings, a putative model of miRNA-target interactions in *O*. *coarctata* under salinity stress was put forward here which proposed various regulation cascades (Fig 9). First, target predictions for differentially expressed salt responsive miRNAs revealed that many targets were TFs including SPB like proteins (*SPLs*), myb domain proteins (*MYBs*), auxin response factor (*ARFs*), APETELA 2 (*AP2*), NAC domain containing proteins (*NACs*), WRKY transcription factors such as WRKY-30, 35,47, 52, 61, 90 and nuclear factor Y subunit (*NF-Y*) (S9 and S10 Tables) which regulate different stress-responsive genes [84]. Many target genes play pivotal roles in plant responses to salinity stress. For an example, miR169-targeted *NF-Y*, which conditions whole plant growth through modifying cell elongation and carbohydrate metabolism, was widely regulated under salinity stress [59]. Moreover, a set of evidences also supported that the involvement of miR164 in stress responses, regulating the miRNA-mediated cleavage of NAC TFs. NACs widely modulate various abiotic stresses including salinity and also integrate responses to environmental stimuli into regulation of plant development processes [65]. Additionally, different members of *ARF* family, which were reported to participate in auxin signalling pathways as negative regulators of growth and development [18], were also identified as salt-responsive e.g. miR160b in radish. Similar findings of miR160-mediated ARF regulation were also reported in salt-stressed *P*. *Tomentosa* [75]. These observations implied that miR160-regulated *ARF*s might contribute to various abiotic stresses. Other genes including miR156/157-targeted *SPLs* and miR172-targeted *AP2*, which might play important role in salinity tolerance, were also identified in this study. It was reported that miR156-regulated *SPLs* and miR172-targeted *AP2* conjointly conditioned the transitions among different developmental stages [85]. Further, miR159-regulated *MYBs* were also considered to modulate plant growth and development especially flowering under salinity stress. It was reported that over-expressed miR159 resulted in a delayed flowering state concomitant with a repression of its target gene, GAMYB in gloxinia [86]. Given that plant growth and development including bolting and flowering are usually adversely-influenced under stress conditions, indicating the involvement of the semi RNA-target transcripts in the network of genes regulated by salt-responsive miRNAs of *O*. *coarctata* (Fig 9).

Secondly, some of the salt-responsive miRNAs target genes involved in protein turnover processes. For an example, ubiqutin-proteosome pathway genes such as F box, cullin-1, RING finger, 4-coumarate—CoA ligase 1 protein were also considered to play important roles in salt stress response [87,88]. It has also been reported that miR399 regulates phosphorus homeostasis by modulating the expression of ubiquitin-conjugating E2 enzyme (UBC) [89].In the present study, oco-miR164e, which targets a ubiquitin family protein that functions in protein degradation, was suppressed upon salt treatment of *O*. *coarctata*. Previous studies have shown that ubiquitin proteins were required for salt tolerance in Arabidopsis by modulating Na^+^ /H^+^ antiporter activity and serine hydroxyl methyltransferase1 (SHM1) stability [90]. The suppression of osa-miR164e in the present study by salt treatment (as indicated by read counts in S3 Table and S4 Table) may result in the increased expression of UBP; this increased expression serves pivotal functions in salt tolerance of *O*. *coarctata*. In Arabidopsis, miR393, which targets F-box proteins, was strongly up-regulated by salt treatment [91]. However, in the present study, the expression of osa-miR393a was down-regulated under salt treatment (as indicated by its read counts in S3 Table and S4 Table); this finding is consistent with other plant species such as *Thellungiella*. *Salsuginea* [92], *Populus*. *Tomentosa* [93] and also in *Salicornia europaea* [22]. Additionally it was reported that ectopic expression of osa-miR393a in rice plants resulted in their more sensitivity to salinity [57]. Therefore, considering our results and those of previous studies mentioned above, we reconfirm that miR393a is a negative regulator of plant salt tolerance including rice.

Furthermore, several target genes were found to be cross-linked by miRNAs in response to salinity stress. They are the genes which basically encode important enzymes or functional proteins, such as peroxidase, glutathione peroxidase, esterase, Ca^2+^-mediated signal-related proteins such as MAP kinase. These genes are well-known for their modulation of abiotic stresses including salinity stress in various plants. Prolonged salinity stress usuallyleads to some secondary stresses such as oxidative and nutrition stresses [90], indicating that the miRNA-regulated target genes involved in these stresses might play significant roles in salinity stress related adaptive responses of plants, as an indispensable part of regulatory network responsive to salinity stress in *O*. *coarctata* (Fig 9).

Thirdly, several miRNAs target genes encoding metabolic enzymes. For an example, inositol-1-monophosphatase (predicted to be target of oco-miR006), glycerol-3-phosphate dehydrogenase (predicted to be target ofoco-miR044) and glucose-6-phosphate isomerise (predicted to be target ofoco-miR005-3p) are involved in sugar metabolism particularly for the metabolism of sorbitol. It was demonstrated that glucose-6-phosphate dehydrogenase is involved in protecting the root cells against oxidative stress in salt-treated red kidney bean.[94]. Therefore, the regulation of glucose-6-phosphate dehydrogenase by oco-miR005-3p may contribute to the maintenance of efficient carbon flux through glycolysis/gluconeogenesis or by sugar alcohol metabolism under salinity stress in *O*. *coarctata*. Similarly inositol-1-monophosphatase gene has been found to be differentially regulated under salinity stress in chickpea and upon ectopic expression, it increased salinity tolerance in chickpea [95]. Therefore, we assume that a similar mechanism might also play an important role for salinity tolerance in *O*. *coarctata*.

Fourth, some specific targets of *O*. *coarctata* miRNAs are related to direct stress responses and the downstream signalling processes. For an example, nucleotide binding site-leucine-rich repeat (NBS-LRR)-type resistance protein which are involved in salinity stress response [96]. In the present study, novel miRNAs such as, oco-miR020-3p andoco-miR026-3p as well as known miRNAs such as oso-miR396c could also target leucine-rich repeat family proteins and phosphoinositide 3-kinase, which are implicated in the signalling pathway.

Finally, many transporters or ion channels were found to be targeted by known and novel miRNAs indicating that these miRNAs were involved in ion homeostasis under salinity stress. Potassium transporter was shown to render the rice plants salinity tolerant by accumulating more K^+^ and less Na^+^ [97]. The potassium transporter (AKT1) and cation transporter (HKT7) which were also found to be predicted targets can modulate monovalent cations and pH homeostasis in plant chloroplast or plastids [98]. Therefore, oco-miR044-3p targeting K^+^ antiporter in the present study may play important roles in the maintenance of Na^+^ and K^+^ homeostasis in *O*. *coarctata*, which is essential for plant survival under saline conditions.

In plants, salinity stress reduces the growth and development initially through osmotic stress and later through salinity stress by increasing cellular Na^+^ and Cl^−^contents which has negative effects on K^+^ and Ca^2+^uptake as well as nutrition [99] indicating that ion homeostasis by ion transporters play crucial role in salinity tolerance. Senadheera et al. [100] reported that cadmium tolerance factor and ABC transporter associated domain containing protein which are also predicted to be the targets in the present study, were up-regulated under salinity stress in the roots of FL-478, a salt tolerant genotype of rice, suggesting that these genes play important role in salinity tolerance. Similarly sulphate transporter which maintains the sulphate flux as sulphate or its derivatives such as glutathione, plays pivotal roles in the defence mechanism of salinity tolerance [101]. In the present study, sulphate transporter which was predicted to be the target of oco-miR044-3p might play similar role in salinity tolerance of *O*. *coractata*.

Therefore, the present results identified known and novel mRNAs of *O*. *coarctata* and also identified differentially expressed miRNAs that are involved in the molecular defence mechanisms of plant response to salinity stress. However, the further study is going on to elucidate the functional relationship of predicted miRNA-target networks.

## Conclusions

In the present study, we profiled miRNAs of *O*. *coarctata* both under control and salt-treated conditions. Besides identifying 338 known and 95 novel miRNAs by using high throughput sequencing, we also identified additional 48 known miRNAs (based on homology search) from publicly available data. Further functional annotation by GO and KEGG pathway analysis indicated that 22 target genes were related to salinity stress response in *O*. *coarctata*. Nine miRNAs such as oco-miR166e-3p, oco-miR169g, oco-miR169o, oco-miR393a, oco-mi396c, oco-miR020-3p, oco-miR014-3p, oco-miR160b and oco-miR395d were found to be salt responsive as indicated by their expressions under salinity stress of *O*. *coarctata*. Additionally, through comparative genomics, we also reported 95 novel miRNAs for the first time in rice and other plants as they are found to be absent in the latest version of miRBase 21 dataset. Some of the targets of these 94 novel miRNAs such as transcription factors and genes coding for enzymes involved in metabolism which may be genetically engineered to generate rice plants with higher salinity tolerance. This would lay the foundation for further research into the molecular mechanisms of salt responsive changes in *O*. *coarctata*.

## Supporting Information

S1 Fig Workflow of small RNA discovery and analysis of *O*. *coarctata*. (DOC)

S2 Fig Annotation and distribution of mRNAs in control (top) and salt-treated library (bottom). (TIF)

S3 Fig Size distribution of small RNA clean reads. (TIF)

S4 Fig Gene Ontology (GO) analysis of salt-stressed target genes of *O*. *coarctata* with AgriGO. Target transcripts of differentially expressed miRNA under salinity stress compared with control were designated as salinity stress responsive genes. Each box shows the GO term number, the p-value in parenthesis, GO term. The first pair of numerals indicates the number of genes in the input list associated with that GO term and the number of genes in the input list. The second pair of numerals depicts the number of genes associated with the particular GO term in the rice database and the total number of rice genes with GO annotations in the rice database. The box colors indicates levels of statistical significance with yellow = 0.05; orange = e-^05^ and red = e-^09^.
(TIF)

S5 Fig Gene Ontology (GO) analysis of salinity stress response target genes of *O*. *coarctata* with REVIGO. (TIF)

S1 Table qRT-PCR primers for miRNAs and their target genes. (DOCX)

S2 Table List of known miRNAs of control library. (XLSX)

S3 Table List of known miRNAs of salt-treated library. (XLSX)

S4 Table List of novel miRNAs of control library. (XLSX)

S5 Table List of novel miRNAs of salt-treated library. (XLSX)

S6 Table List of the miRNAs discovered through *in silico* analysis. (XLSX)

S7 Table Differentially expressed known miRNAs between control and salt-treated library. (XLS)

S8 Table Differentially expressed novel miRNAs between control and salt-treated library. (XLS)

S9 Table Predicted targets of differentially expressed known miRNAs between control and salt-treated library. (XLSX)

S10 Table Predicted targets of differentially expressed novel miRNAs between control and salt-treated library. (XLSX)

S11 Table Enriched GO categories of the salinity stress repressed transcripts in *O*. *coarctata*. (XLSX)

S12 Table The miRNA targets involved in *O*. *coarctata* salinity stress response according to a KEGG pathway analysis. The homologs of rice gene orthologues are given.
(XLSX)

S13 Table Novel miRNAs in selected monocot and dicot plants. (XLSX)
