# Supplementary material for: Identification of Novel and Conserved miRNAs from Extreme Halophyte, Oryza coarctata, a Wild Relative of Rice
Source: PLoS One. 2015 Oct 27;10(10):e0140675. doi: 10.1371/journal.pone.0140675 (PMC4623511; doi:10.1371/journal.pone.0140675)
Supplement: S1 Fig — (DOC) [file pone.0140675.s001.doc]

Extraction of total RNA

Isolation of small RNA

Construction of smallRNA library

construction

Sequencing of small RNA library with Illumina Hiseq platform

Quality control of raw read

construction

Adaptor trimming

Reads with length <16 and >36 nt removed

Reads matching to tRNA,rRNA, intron, exon etc were excluded

Mapping the reads to miRBase (*Oryza saitiva*)

Identification of known miRNAs that mapped to the miRBase

Balance reads not showing match to the miRBase

Determining read count

Prediction of novel miRNAs and determination of read count

Identification of differentially expressed known miRNA

Identification of differentially expressed novel miRNA

miRNA

Target prediction and gene ontology determination

Target prediction and gene ontology determination

**Supplementary Fig S1 Workflow of small RNA discovery and analysis of *O. coarctata***
