# Supplementary figures and images for: Identification of Novel and Conserved miRNAs from Extreme Halophyte, Oryza coarctata, a Wild Relative of Rice
Source: PLoS One. 2015 Oct 27;10(10):e0140675. doi: 10.1371/journal.pone.0140675 (PMC4623511; doi:10.1371/journal.pone.0140675)

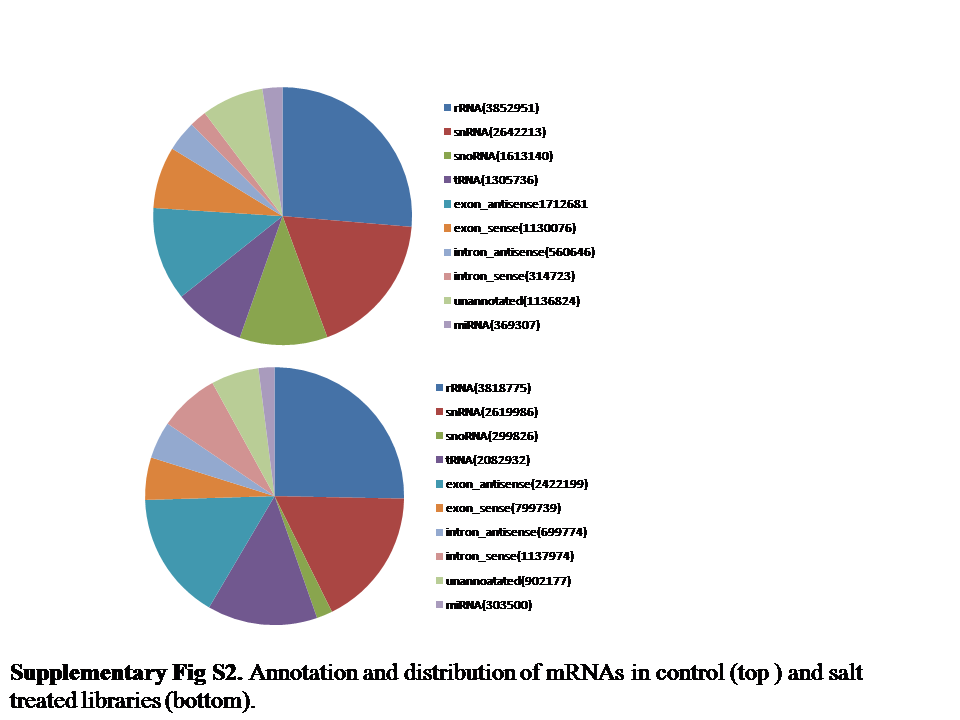

Supplement: S2 Fig — (TIF) [file pone.0140675.s002.TIF]

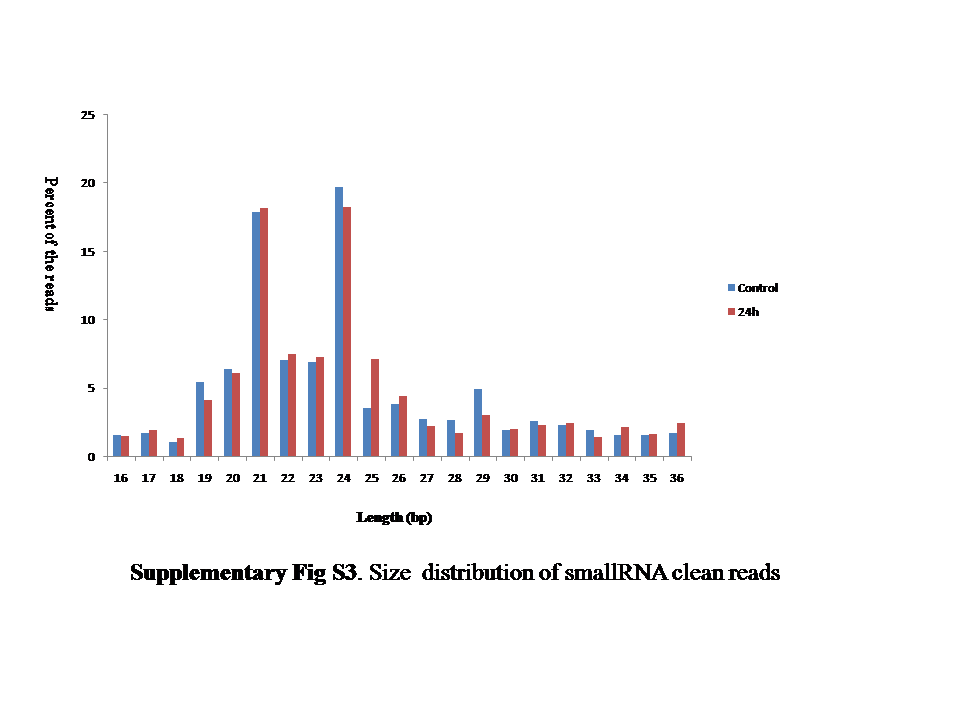

Supplement: S3 Fig — (TIF) [file pone.0140675.s003.TIF]

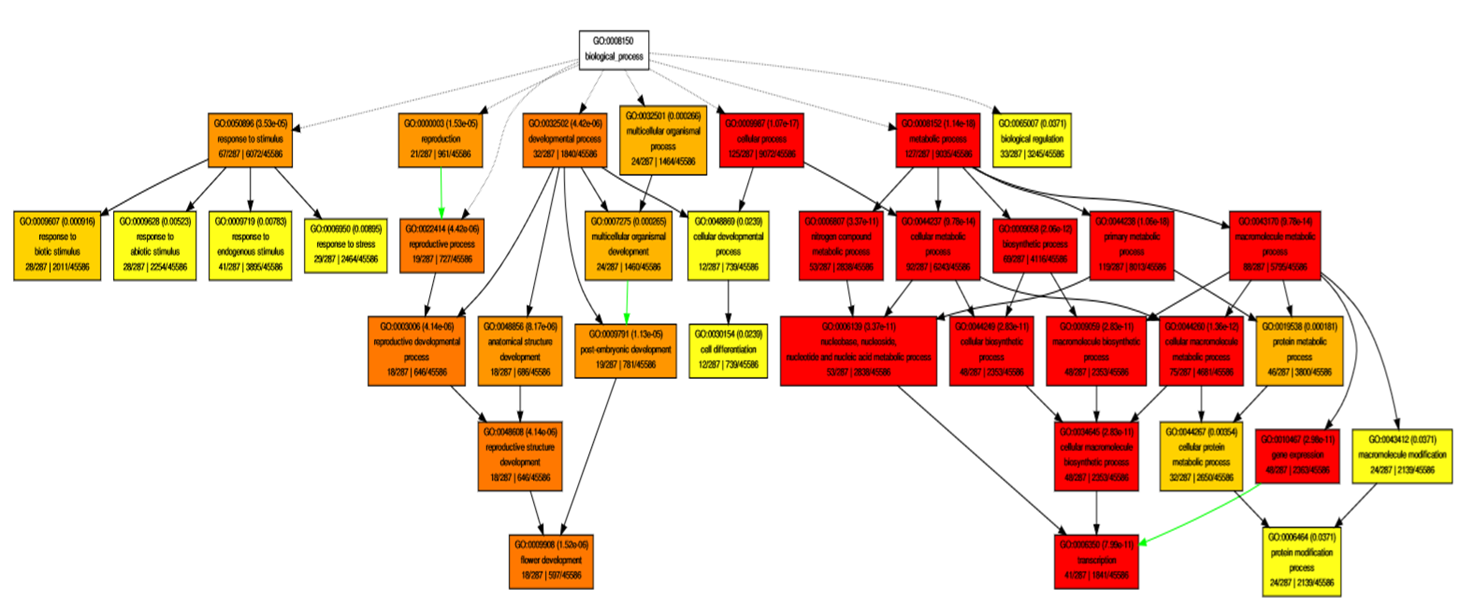

Supplement: S4 Fig — Target transcripts of differentially expressed miRNA under salinity stress compared with control were designated as salinity stress responsive genes. Each box shows the GO term number, the p-value in parenthesis, GO term. The first pair of numerals indicates the number of genes in the input list associated with that GO term and the number of genes in the input list. The second pair of numerals depicts the number of genes associated with the particular GO term in the rice database and the total number of rice genes with GO annotations in the rice database. The box colors indicates levels of statistical significance with yellow = 0.05; orange = e-05 and red = e-09. (TIF) [file pone.0140675.s004.TIF]

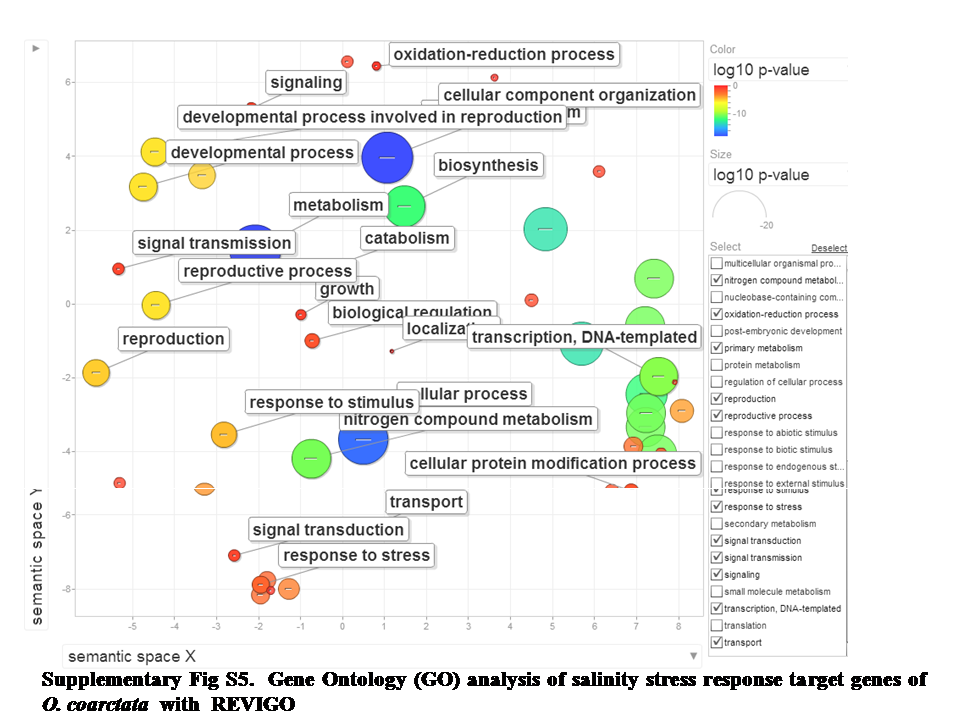

Supplement: S5 Fig — (TIF) [file pone.0140675.s005.TIF]
