# Supplementary material for: Identification of Novel and Conserved miRNAs from Extreme Halophyte, Oryza coarctata, a Wild Relative of Rice
Source: PLoS One. 2015 Oct 27;10(10):e0140675. doi: 10.1371/journal.pone.0140675 (PMC4623511; doi:10.1371/journal.pone.0140675)
Supplement: S1 Table — (DOCX) [file pone.0140675.s006.docx]

Supplementary Table S1: Primers used in the present study.

| miRNA | Nature | Regulation as solexa analysis | miRNA specific forward primer | Target ID | Target specific forward primer (5’-3’) | Target specific reverse primer (5’-3’) |
| --- | --- | --- | --- | --- | --- | --- |
| oco-miR166e-3p | Known | Up | TCGAACCAGGCTTCATTCCCC | LOC_Os08g34740 | GATGAAAATTGCTTGTGGGTTTGAGATG | ATCTCCCTTGTTCGTGAAAACAAACTCG |
| oco-miR169g | Known | Up | TAGCCAAGGATGACTTGCCTA | LOC_Os02g53620 | CCTCATCGTCTTCTTATTCCTACGCCTC | GATGCCCAAACTCCTGAACCAGAATC |
| oco-miR169o | Known | Up | TAGCCAAGAATGACTTGCCTA | LOC_Os03g48970 | GATGACTGCTTTGGCATCAGACTATTTAACAC | CATGATATTGCTTAGCATTTACATAAACAGGC |
| oco-miR393a | Known | Down | TCCAAAGGGATCGCATTGATC | LOC_Os05g41010 | AGGGTGTTCCTCTACCAGCATTTCCTC | CTCTTTAGGAAGAAATGTCCACGCAGC |
| oco-miR396c | Known | Down | TTCCACAGCTTTCTTGAACTT | LOC_Os05g10580 | GCAATTTTCTTCTTGCAGAGATGCTGC | CAGGAGTGCACGGTCTATGAGCAAAC |
| oco-miR10-5p | Novel | Down | CACCGCAGTAGAGTTTAACCACC | LOC_Os05g41172 | TCTAGGGTTTCCACCACCTCTCCACTC | GGGTGCTTGACTGGTTGACATTGATT |
| oco-miR020-3p | Novel | Up | CACCGCAGTAGAGTTTAACCACC | LOC_Os06g16790 | CCTAGGTAACATAGAGAAAATTATTAACG | AATAATTTAGAGCACTGC TGGAGTTTG |
| oco-miR028-5p | Novel | Up | ATGTGTACACTTGTAGTAGCCA | LOC_Os11g02470 | ACA AGT CGT CGT CAT GGT CTC AAT TC | GGTGGGTCGTTCTTATCCTTTTGTTG |
| oco-miR044-3p | Novel | Down | CCGCGCCGCCGCCGGCCGCC | LOC_Os07g02330 | GGT CAT GGC GTC GCC CGG CGC CAC | TTCGGCAGCGACCACCGCAGCAGCTG |
| oco-miR014-3p | Novel | Down | AGAGTTATGAAGGTGTGGCA | LOC_Os02g49520 | AAT CCG ATG AAT TTG GAG GCC ACT TGT TC | GACATTTTCAGAACTATCAGCAAACCTCTC |
| oco-miR156d | *In silico* | NA | TGACAGAAGAGAGTGAGCAC | LOC_Os11g30370 | TTCACAATCTGGCTGAGTTTGACGATG | AGAATGAGATGGAGAAGGGAGGGTGAC |
| oco-miR160b | *In silico* | NA | TGCCTGGCTCCCTGTATGCCA | LOC_Os04g43910 | GACAGTAGTGCTCAACCTGCAGGCATAC | AGAATTGCCTGTCCAAAGAGGAGAAACC |
| oco-miR162b | *In silico* | NA | TCGATAAGCCTCTGCATCCAG | LOC_Os03g56370 | GAGTGCACGGTATTTGAACTTGACTTCC | CCTTTCAGGAACATAGCCGTAGCTCTGTAG |
| oco-miR166g | *In silico* | NA | AATGGAGGCTGATCCAAGATC | LOC_Os01g55580 | CAAGCACTCCAAATGTTTGAAGTTGTATTG | CAAGCACTCCAAATGTTTGAAGTTGTATTG |
| oco-395d | *In silico* | NA | GTGAAGTGTTTGGGGGAACTC | LOC_Os03g09940 | TCTTCCAGGTCTCCTTCGGCTTGTTC | TGGCGAGGATGAAGATGAGGAATGAG |
| Outer primer for 5’RACE-PCR | | | CTGAAGACTCTTAAGCTCAACAATG | TIR1 orthologue from *O. coartata* | | |
| Nested primer for 5’ RACE-PCR | | | ACTTAATCTGAGTTATGCTACTGTGC | TIR1 orthologue from *O. coartata* | | |
| NA= Not available | | | | | | |
